# Impact of social vulnerability on frailty transition among older adults in China: a national two-year cohort study

**DOI:** 10.7189/jogh.15.04284

**Published:** 2025-11-07

**Authors:** Jing Shi, Yongkang Tao, Yan Cen, Chao Gao, Luyao Zhang, Sainan Li, Ying Li, Botao Sang, Xiangfei Liu, Qinan Ma, Xuezai Zeng, Jing Li, Deping Liu

**Affiliations:** 1 The Key Laboratory of Geriatrics, Beijing Institute of Geriatrics, Institute of Geriatric Medicine, Chinese Academy of Medical Sciences, Beijing Hospital/National Center of Gerontology of National Health Commission, Beijing, China; 2 Department of Gastroenterology, China-Japan Friendship Hospital, Beijing, China; 3 Department of Cardiology, Beijing Hospital, National Center of Gerontology, Institute of Geriatric Medicine, Chinese Academy of Medical Sciences, Beijing, China; 4 Peking Union Medical College, Chinese Academy of Medical Sciences, Graduate School of Peking Union Medical College, Beijing, China; 5 Peking University Fifth School of Clinical Medicine, Beijing, China; 6 Savaid Medical School, University of Chinese Academy of Sciences, Beijing, China; 7 Department of Geriatric Medicine, Beijing Hospital, National Center of Gerontology, Institute of Geriatric Medicine, Chinese Academy of Medical Sciences, Beijing, China

## Abstract

**Background:**

Frailty in older adults has become a major concern. It is influenced by biological, social, psychological, and environmental factors, with social frailty playing a particularly significant role. The relationship between social frailty and health outcomes can accelerate frailty transitions, offering new insights into strategies to improve health in older adults.

**Methods:**

We obtained the data from the Fourth Sample Survey of the Aged Population in Urban and Rural China, with 2017 as the baseline and 2019 as the follow-up. We used the frailty index (FI) to assess physiological frailty and the social vulnerability index (SVI) to assess social frailty. We used logistic regression to analyse the impact of SVI on frailty transitions.

**Results:**

The analysis included 9093 older individuals, with an average age of 71.2 (standard deviation = 7.0) years, comprising 4495 women and 4598 men. Of these, 39.3% were robust, 45.1% were prefrail, and 15.6% were frail. Both the FI and SVI increased with age, and women showed higher frailty levels. Over the two years, 56.2% maintained stable frailty status, 14.2% improved, and 29.6% deteriorated. Correlation analysis revealed a moderate relationship between FI and SVI (*r* = 0.337; *P* < 0.001). Logistic regression analysis indicated that higher social frailty was associated with an increased risk of worsening frailty in non-frail (odds ratio (OR) = 1.017; *P* < 0.05) and prefrail individuals (OR = 1.021; *P* < 0.05), but had no effect on those who were already frail.

**Conclusions:**

Frailty deterioration with age is more common than improvement. Increased social frailty is a significant risk factor for the worsening of frailty, particularly in non-frail and prefrail individuals. Early identification and prevention of social frailty may help delay its progression.

Studying frailty in older adults has become a significant focus in health and ageing research. Frailty is a complex, dynamic physiological condition characterised by a decline in bodily functions and physical capabilities. It has emerged as one of the most common health challenges among older adults, leading to adverse health events such as falls, disability, mortality, and impairment in activities of daily living (ADL) and instrumental ADL, which collectively impose a considerable economic and caregiving burden on families and society globally [1–3]. As a result, frailty represents a critical concern for public health and medical systems. The integrative model of frailty identifies three distinct stages: non-frail (robust), prefrail, and frail [4]. Frailty transitions, which involve either deterioration or improvement, are not only influenced by biological factors but also by social, psychological, and environmental variables, with social frailty playing a crucial role [5–8].

In recent years, the concept of social frailty has garnered increasing attention. While social frailty is important across all age groups, its impact is particularly significant for individuals aged ≥60, given the higher incidence of major social changes that can lead to feelings of social isolation in later life [9]. These changes include retirement, death of a life partner, and growing dependence on others for ADL, such as shopping or personal hygiene [10,11]. For some older adults, the accumulation of these social changes can contribute to declines in functional, psychological, and physiological well-being [11] – factors commonly recognised as components of frailty [12]. Understanding the factors that influence frailty and its transitions, particularly in relation to social frailty, offers a valuable new perspective on improving the health and well-being of older adults.

While much of the existing research on frailty in older adults has focused on physiological factors, the role of social frailty, particularly in shaping frailty transitions, is relatively underexplored, especially within the Chinese context. Also, the social vulnerability index (SVI), a comprehensive indicator of social frailty, is effective and statistically robust across cultures and environmental contexts [13], but its application for assessing social frailty among older adults in China remains relatively limited. We aim to address this gap by using data from the Fourth Sample Survey of the Aged Population in Urban and Rural China (SSAPUR), the largest database of older adults in China to date. Meanwhile, we applied both the frailty index (FI) and the SVI to comprehensively assess physiological and social frailty. We aimed to comprehensively analyse social frailty among Chinese older adults and to investigate its impact on transitions between different states of physiological frailty. We offer novel insights by exploring how social vulnerability – including economic status, social engagement, and family support – impacts frailty progression among older adults in China, a nation with unique socioeconomic and health care challenges. By examining these factors, we offer new perspectives on frailty transitions, particularly in the context of China’s ageing population, and propose targeted interventions to mitigate the risk of frailty progression.

## METHODS

### Study design and participants

We obtained the data from the Fourth SSAPUR database, organised by the China National Committee on Aging. This survey was a comprehensive, large-scale nationwide study conducted among residents aged ≥60 in mainland China. The survey was initiated from 1 August to 31 August 2015 and was followed by continuous monitoring conducted by the China National Committee on Aging in 2016, 2017, 2018, and 2019 to dynamically assess the living conditions and health status of the older adults in the 2015 survey sample. To ensure the representativeness of the sample, the survey utilised a stratified, multi-stage, probability-proportional-to-size sampling method. The sample size was set at 223 680, corresponding to a sampling ratio of approximately 0.1%, based on the proportion of the population aged ≥60 in the survey areas. The sampling process consisted of four steps, and the survey sample structure and methodology have been detailed in previous research [14,15]. The survey encompassed all provinces, autonomous regions, municipalities, and the Xinjiang Production and Construction Corps, covering 466 counties (districts), 1864 townships (subdistricts), and 7456 villages (residential committees). After excluding individuals who refused to participate, were deceased, could not be contacted (despite at least three attempts), or were long-term residents of nursing institutions during the survey period, the final sample size was 224 142. This makes the database the largest one for the older population in China to date. All participants gave written informed consent before completing the survey.

Since the 2017 survey population overlaps more with the 2019 cohort, we used the 2017 data as the baseline and the 2019 data as the follow-up to analyse changes in the physiological frailty status of the older population from 2017 to 2019. Of the 12 788 older adults assessed in 2017, 3695 were lost to follow-up by 2019, leaving a final sample of 9093 older individuals. A comparison of the general characteristics between the follow-up cohort and those lost to follow-up revealed that the latter were slightly older (mean (x̄) = 73.0, standard deviation (SD) = 8.0 years *vs.* x̄ = 71.2, SD = 7.0 years; *P* < 0.05), had higher FI (x̄ = 0.16, SD = 0.11 *vs.* x̄ = 0.14, SD = 0.09; *P* < 0.05), and SVI values (x̄ = 0.42, SD = 0.12 *vs.* x̄ = 0.40, SD = 0.12; *P* < 0.05).

### Survey content

The survey data were collected through household interviews and questionnaires, covering a range of topics including demographic characteristics (*e.g.* age, gender, ethnicity, educational level, and marital status), family status (*e.g.* living alone, and children’s situation), health and medical conditions (*e.g.* vision, hearing, exercise habits, physical examinations, chronic diseases, medical visits, medical insurance status, and self-assessed health status), care and nursing services (*e.g.* ADL, incontinence, use of assistive devices, care conditions, and willingness for care), economic status (*e.g.* income, expenditures, and homeownership), living environment (*e.g.* housing area, conditions, and satisfaction with living conditions), social participation (*e.g.* involvement in public welfare activities, and willingness to assist other older adults), rights protection (*e.g.* awareness of rights and legal protection), and cultural and mental well-being (*e.g.* participation in community activities and frequent internet use). The diseases included required a diagnosis from a hospital at the county level or higher, whereas conditions with symptoms but no confirmed diagnosis were excluded.

### Frailty assessment and FI transition

Based on the survey questionnaire, the frailty assessment utilised the FI model developed by Professor Kenneth Rockwood's team. This method quantifies an individual’s degree of frailty based on the accumulation of health deficits. Following the standard procedures for constructing the FI and the criteria that health deficit variables must meet, a total of 31 variables were selected to calculate the FI [16]. The variables were assigned values according to their type, following standard procedures. The specific 31 variables include ADL (six items), chronic diseases (11 items), geriatric syndromes (five items), health status and emotions (four items), and assistive device use (five items) (Table S1 in the **Online Supplementary Document**). The FI is calculated using the formula: FI = number of health deficits present / total number of items considered as health deficits (in this case, 31), with a value range from 0 to 1. A higher FI value indicates a greater number of health deficits and, consequently, greater frailty. Based on existing research [17,18], we categorised frailty into three levels: robust (FI≤0.10), prefrail (FI = 0.10–0.21), and frail (FI>0.21). We defined a transition in frailty status over two years as a change among the three frailty states (robust, prefrail, and frail), encompassing deterioration, stability, or improvement.

### Social frailty

Social frailty was assessed using the SVI, which is based on the survey questionnaire. Previous studies have demonstrated the effectiveness of the SVI in evaluating social vulnerability across various contexts [10,19,20]. Rather than focusing on individual factors, the SVI quantifies social frailty by considering multiple facets of social circumstances. Using the SVI to reflect an individual’s social characteristics reduces dimensionality, enabling the inclusion of substantial information in statistical models without the need for many separate parameters, which could otherwise lead to multicollinearity and model instability. The variables used to construct the SVI should meet two criteria. First, they should broadly capture an individual's social environment, including economic status, living conditions, social participation, and social support. Second, these factors should also affect health status [21]. Ultimately, we selected 21 social vulnerability variables (Table S2 in the **Online Supplementary Document**). The assignment of values followed a method similar to that used for the FI. For binary variables, the presence of a social deficit was assigned a value of 1, and its absence a value of 0. For ordinal variables, intermediate values were assigned. For example, in the economic status variable, ‘very wealthy’ was assigned a value of 0, ‘fairly wealthy’ 0.25, ‘just enough’ 0.50, ‘somewhat difficult’ 0.75, and ‘very difficult’ a value of 1. Thus, each variable's social frailty score was assigned a value between 0 and 1. For each individual, we calculated the SVI by summing the assigned scores and dividing by the total number of items considered as social deficits. The SVI ranged from 0 to 1, with higher values indicating greater social frailty [21].

### Missing data and sensitivity analysis

All missing data occurred exclusively in the constituent items of the FI and SVI. Specifically, among the 31 FI components and 21 SVI indicators analysed, the overall missing data rate was 8.92%, with variable-specific rates ranging 0.05–4.87% for FI items and 0.12–15.00% for SVI indicators. Since the variables with missing data were continuous, we used the multiple imputation by chained equations method with Markov chain Monte Carlo sampling, specifying ten imputations and five iterations per chain.[22].

Although we employed multiple imputation by chained equations under the missing-at-random assumption for our primary analysis, we conducted comprehensive sensitivity analyses to evaluate potential bias from missing-not-at-random mechanisms. Based on observed differences showing that dropouts exhibited significantly higher baseline FI (>0.02) and SVI (>0.02) scores than retained participants, we implemented a deterministic sensitivity analysis in three sequential steps [23]. First, we identified high-risk participants among those retained in follow-up who had baseline values matching or exceeding the dropout group means (FI≥0.16 or SVI≥0.42). Second, we systematically augmented these participants' index scores by empirically derived increments (ΔFI>0.02; ΔSVI>0.02) to simulate the potential missing-not-at-random scenario where these high-risk individuals might have been lost to follow-up. Finally, we re-estimated the primary regression models using these adjusted values to assess the robustness of our findings under plausible missing-not-at-random conditions.

### Data analysis

We used SPSS, version 24.0 (IBM Corp., New York, USA) and *R*, version 4.3.2 (R Core Team, Vienna, Austria) for all analyses. We presented continuous variables as x̄ (SD), and used independent-samples *t* tests for comparisons between two groups and analysis of variance for comparisons among multiple groups. We presented categorical variables as numbers (percentages), and used the χ^2^ (χ*^2^*) test for comparisons between groups. We applied nonlinear regression techniques to fit age-specific FI values as a function of age (using an exponential function) for both baseline and follow-up, and to compare older adults of different genders. We used histograms to illustrate the distribution of the SVI and bar graphs to compare the SVI across age groups of men and women. We used Pearson correlation analysis to assess the relationship between FI and SVI, and analysed changes in frailty status among older adults with varying baseline frailty levels. We employed linear regression to model the x̄ SVI as a function of age. We conducted binary and multinomial logistic regression analyses across baseline frailty levels to examine the impact of the SVI on the transition in frailty status among older adults. We assessed multicollinearity by calculating variance inflation factors (VIFs) and tolerance values for each predictor using auxiliary linear regressions, with thresholds of >2.5 and <0.1, respectively. We evaluated the potential interaction effects among the independent variables by including multiplicative terms in the model. We considered a *P*-value <0.05 as statistically significant.

## RESULTS

### Comparison of general characteristics and social frailty-related factors across different frailty conditions in older adults

Among the 9093 older adults, ages ranged from 62 to 101 years (x̄ = 71.2, SD = 7.0), of which 4495 were women (x̄ = 71.5, SD = 7.2 years) and 4598 men (x̄ = 70.9, SD = 6.8 years) (Table S2 in the **Online Supplementary Document**). There were 3572 (39.3%) participants classified as robust, 4098 (45.1%) as prefrail, and 1423 (15.6%) as frail. Adults who were older, female, from ethnic minorities, rural residents, or had lower education levels had higher proportions of prefrailty and frailty. Ethnicity and place of residence had no statistically significant effect on frailty in older men, while age and education level remained significant factors. For older women, age (*P* < 0.001), ethnicity (*P* = 0.017), place of residence (*P* < 0.001), and education level (*P* < 0.001) all had statistically significant effects on their frailty status. Regarding social frailty-related factors, all factors – except medicare reimbursement, private living rooms, and participation in online education – were found to influence frailty levels in both men and women. The average SVI was 0.40 (SD = 0.12), with both older men and women showing an increase in frailty levels as SVI increased. When stratifying social frailty levels among older adults by SVI quartiles, the results indicated that frailty levels increased with greater social frailty, regardless of whether the analysis included the entire population, older men, or older women.

### Analysis of frailty and social frailty conditions in older adults

**Figure 1:**
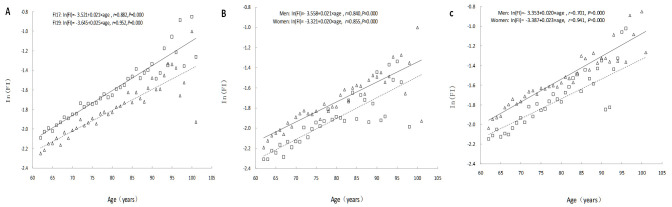
The relationship between age and FI. **Panel A.** Comparison of older adults at baseline in 2017 and follow-up in 2019. **Panel B.** Comparison of older adults by gender at baseline in 2017. **Panel C.** Comparison of older adults by gender at follow-up in 2019. FI – frailty index.

At baseline in 2017, the 9093 older participants had an FI ranging from 0 to 0.76 (x̄ = 0.14, SD = 0.09), with females having a slightly higher FI (x̄ = 0.15, SD = 0.09) than males (x̄ = 0.12, SD = 0.08). At the 2019 follow-up, FI values ranged from 0 to 0.77 (x̄ = 0.16, SD = 0.11), with females still exhibiting a slightly higher score (x̄ = 0.18, SD = 0.11) than males (x̄ = 0.15, SD = 0.10). An analysis of the trend in FI values with age revealed an exponential increase, described by the equation ln(FI) = A + B × age. The FI value in 2019 was higher than in 2017, and the average annual relative FI growth rate in 2019 (B = 0.025) exceeded that of 2017 (B = 0.021) (*t =* 7.737, *P* < 0.001) (**Figure 1**, Panel A). Further gender-specific analysis revealed that, at every age, FI values for females were consistently higher than those for males, indicating greater frailty in older women. However, in 2017, the average annual relative FI growth rate for older women (B = 0.020) was slightly lower than that for men (B = 0.021), although the difference was not statistically significant (*t =* 1.049; *P* = 0.206). In contrast, in 2019, the average annual relative FI growth rate for women (B = 0.023) was higher than that for men (B = 0.020) (*t =* 5.100; *P* < 0.001), suggesting that older women accumulate health deficits at a faster rate than men (**Figure 1**, Panels B and C).

**Figure 2:**
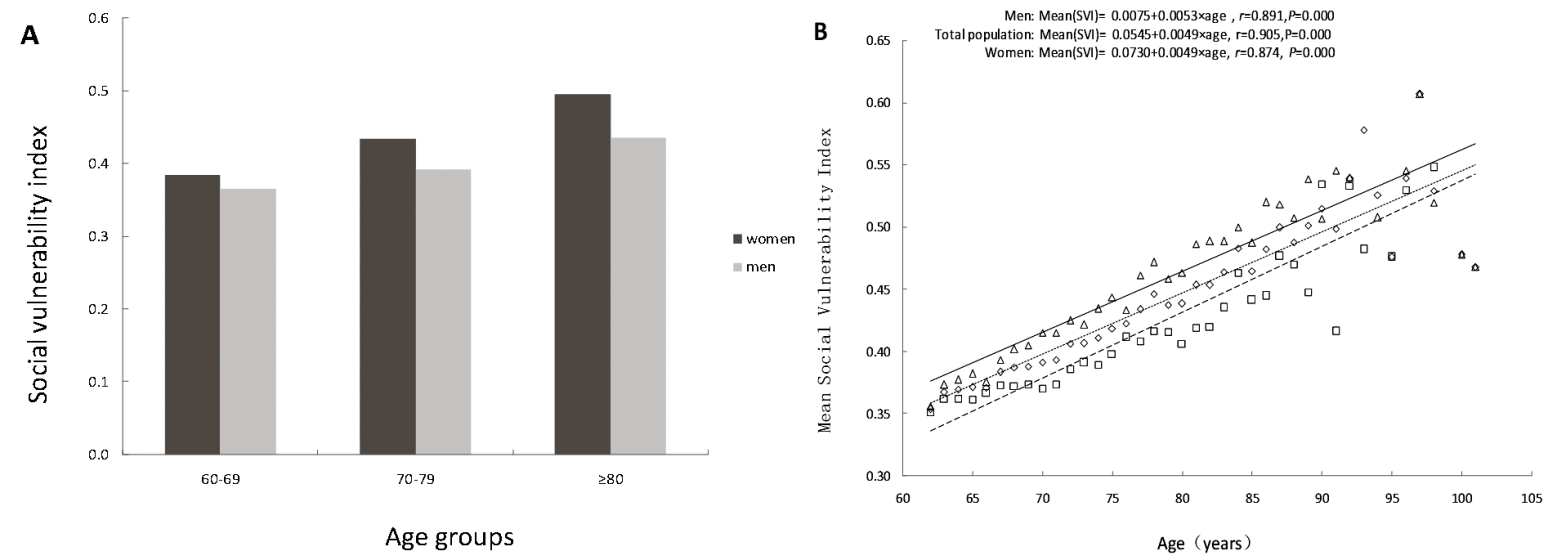
SVI by age and sex. **Panel A.** SVI among different age groups. **Panel B.** The relationship between age and SVI. SVI – social vulnerability index.

At baseline in 2017, SVI values ranged from 0.05 to 0.85 (x̄ = 0.40, SD = 0.12). While some older individuals did not exhibit frailty (FI = 0), all demonstrated some degree of social vulnerability (SVI>0) (Figure S1, Panel A in the **Online Supplementary Document**). Females had a slightly higher SVI score (x̄ = 0.42, SD = 0.12) than males (x̄ = 0.38, SD = 0.11), with similar distributions of SVI in both sexes (Figure S1, Panels B and C in the **Online Supplementary Document**). Further analysis of the trend in SVI with age revealed that SVI increased with age. At every age, females had higher SVI values than males, indicating greater social vulnerability in older women. However, the average annual relative SVI growth rate for women (B = 0.049) was lower than that for men (B = 0.053) (*t =* 4.695; *P* < 0.001), suggesting that social frailty progresses more rapidly in older men than in women (**Figure 2**, Panels A and B). Correlation analysis between FI and SVI revealed a moderate relationship between social frailty and physical frailty in older adults (*r =* 0.337; *P* < 0.001), with the correlation being stronger in women (*r =* 0.330; *P* < 0.001) than in men (*r* = 0.316; *P* < 0.001).

### Changes in frailty status among older adults

At baseline in 2017, there were 3572 (39.3%) robust, 4098 (45.1%) prefrail, and 1423 (15.6%) frail individuals. By 2019, there were 2784 (30.6%) robust, 4089 (45.0%) prefrail, and 2220 (24.4%) frail individuals. Over the two-year follow-up, 5111 (56.2%) individuals maintained stable frailty, 1292 (14.2%) showed improvement, and 2690 (29.6%) experienced deterioration. Specifically, 54.3% (n/N = 1939/3572) of robust, 55.1% (n/N = 2259/4098) of prefrail, and 64.2% (n/N = 913/1423) of frail individuals maintained their baseline frailty status. In terms of frailty progression, 38.7% (n/N = 1383/3572) of robust individuals transitioned to prefrail, 25.8% (n/N = 1057/4098) of prefrail individuals transitioned to frail, and 7.0% (n/N = 250/3572) of robust individuals transitioned directly to frail. Notably, 31.4% (n/N = 447/1423) of frail individuals improved to prefrail status, 19.1% (n/N = 782/4098) of prefrail individuals improved to robust, and 4.4% (n/N = 63/1423) of frail individuals reverted to a robust state over the two years. Overall, transitions to a more severe state of frailty (*i.e.* deterioration) were more common than improvements, and transitions between adjacent frailty states (n = 3669; 40.3%) were more frequent than those spanning multiple states (n = 313; 3.4%) (Figure S2 in the **Online Supplementary Document**).

**Figure 3:**
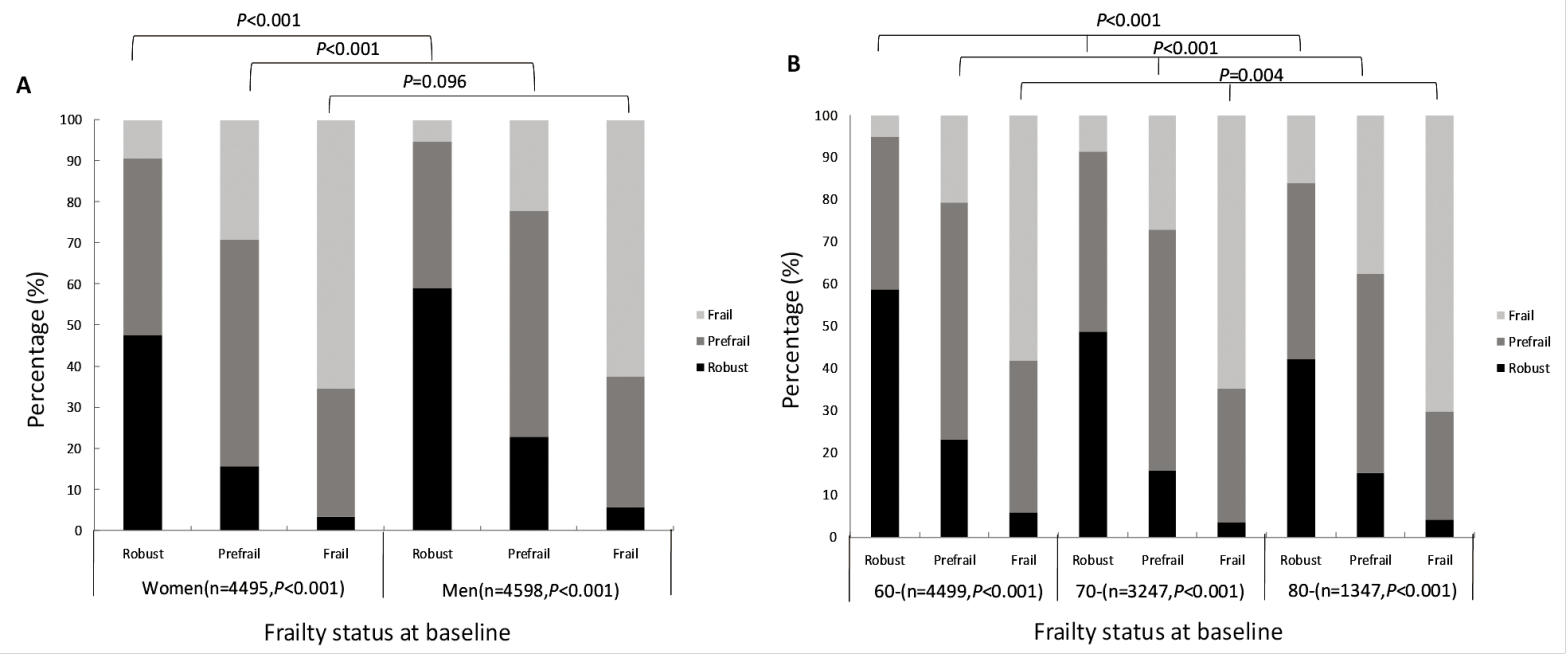
Development of frailty status from baseline to follow-up. **Panel A.** By gender. **Panel B.** By age group.

Further analysis by gender revealed that among 4495 older women, 2453 (54.6%) maintained stable frailty status, 635 (14.1%) improved, and 1407 (31.3%) experienced deterioration. Among 4598 older men, 2658 (57.8%) remained stable, 657 (14.3%) improved, and 1283 (27.9%) deteriorated. A higher proportion of women experienced worsening frailty than men (χ^2^ = 13.148; *P* = 0.001). A higher proportion of men remained stable or improved compared to women among robust (χ^2^ = 55.849; *P* < 0.001) and prefrail individuals (χ^2^ = 47.820; *P* < 0.001). However, for those already frail, the difference in transitions between men and women was not statistically significant (χ^2^ = 4.695; *P* = 0.096) (**Figure 3**, Panel A; **Table 1**). Among the 4499 individuals in the 60–69 age group, frailty status remained stable in 2595 (57.7%), improved in 623 (13.8%), and worsened in 1281 (28.5%). For the 3247 individuals in the 70–79 age group, frailty status remained stable in 1806 (55.6%), improved in 454 (14.0%), and worsened in 987 (30.4%). Lastly, for the 1347 individuals in the ≥80 age group, frailty status remained stable in 710 (52.7%), improved in 215 (16.0%), and worsened in 422 (31.3%). An increase in age was associated with a higher risk of frailty deterioration (χ^2^ = 12.297; *P* = 0.015). The association between age and changes in frailty was significant, regardless of whether individuals were robust (χ^2^ = 82.809; *P* < 0.05), prefrail (χ^2^ = 93.467; *P* < 0.05), or frail (χ^2^ = 15.251; *P* < 0.05) at baseline. This indicates that with advancing age, individuals are more likely to transition towards a state of frailty (**Figure 3**, Panel B).

**Table 1:** Development of frailty status at two-year follow-up according to gender and age

	Gender				Age group				
	Women*	Men*	***χ^2^***	***P*-value**	60–69*	70–79*	≥80*	***χ^2^***	***P*-value**
**Total, n**	4495	4598			4499	3247	1347		
**Robust to**	1481	2091	55.849	<0.001	2181	1084	307	82.809	<0.001
Robust	703 (47.5)	1236 (59.1)			1281 (58.7)	528 (48.7)	130 (42.3)		
Prefrail	638 (43.1)	745 (35.6)			792 (36.3)	463 (42.7)	128 (41.7)		
Frail	140 (9.4)	110 (5.3)			108 (5.0)	93 (8.6)	49 (16.0)		
**Prefrail to**	2157	1941	47.820	<0.001	1854	1590	654	93.467	<0.001
Robust	338 (15.7)	444 (22.9)			429 (23.1)	253 (15.9)	100 (15.3)		
Prefrail	1190 (55.2)	1069 (55.1)			1044 (56.3)	906 (57.0)	309 (47.2)		
Frail	629 (29.1)	428 (22.0)			381 (20.6)	431 (27.1)	245 (37.5)		
**Frail to**	857	566	4.695	0.096	464	573	386	15.251	0.004
Robust	30 (3.5)	33 (5.8)			27 (5.8)	20 (3.5)	16 (4.1)		
Prefrail	267 (31.2)	180 (31.8)			167 (36.0)	181 (31.6)	99 (25.6)		
Frail	560 (65.3)	353 (62.4)			270 (58.2)	372 (64.9)	271 (70.2)		

χ^2^ – chi square

*All values are presented as numbers (percentages)

### Univariate and multivariate analysis of the impact of SVI on frailty transitions in older adults

Univariate analysis revealed that the differences in age, gender, ethnicity, place of residence, SVI, and baseline FI among older individuals with different frailty transition statuses were all statistically significant (**Table 2**). Compared to individuals with stable frailty conditions, those whose conditions deteriorated were older. Women were more likely to experience worsening frailty than men. Additionally, ethnic minorities showed a higher proportion of frailty improvement than the Han Chinese. Furthermore, rural residents were more prone to frailty deterioration than urban residents. Higher SVI levels were associated with a greater tendency toward frailty, and older individuals with higher baseline frailty levels were more likely to show improvement in their frailty status.

**Table 2:** Determinants of the development of frailty status from baseline to follow-up

	Stability*	Worsening*	Improvement*	χ^2^	*P*-value
**Total, n**	5111	2690	1292		
**Age, x̄ (SD)**	71.0 (6.9)	71.5 (7.0)	70.1 (7.2)	6.617†	0.001
**Age group**				16.864	0.002
60–69	2594 (57.7)	1254 (27.9)	651 (14.4)		
70–79	1805 (55.6)	986 (30.4)	456 (14.0)		
≥80	712 (52.9)	450 (33.4)	185 (13.7)		
**Gender**				13.148	0.001
Female	2453 (54.6)	1407 (31.3)	635 (14.1)		
Male	2658 (57.8)	1283 (27.9)	657 (14.3)		
**Ethnicity**				7.975	0.019
Han	4907 (56.3)	2592 (29.7)	1221 (14.0)		
Minority	204 (54.7)	98 (26.3)	71 (19.0)		
**Residence place**				38.747	<0.001
Urban	2727 (58.6)	1242 (26.7)	687 (14.8)		
Rural	2384 (53.7)	1448 (32.6)	605 (13.7)		
**Education**				8.944	0.063
No formal education	3758 (56.7)	1958 (29.5)	917 (13.8)		
≤Junior high school	1250 (55.0)	686 (30.2)	337 (14.8)		
≥Senior high school	103 (55.1)	46 (24.6)	38 (20.3)		
**SVI, x̄ (SD)**	0.40 (0.12)	0.43 (0.12)	0.40 (0.11)	31.848†	<0.001
**SVI level**				18.858	0.004
Q1	1366 (59.0)	617 (26.7)	331 (14.3)		
Q2	1303 (57.1)	666 (29.2)	311 (13.6)		
Q3	1184 (53.8)	693 (31.5)	323 (14.7)		
Q4	1258 (54.7)	714 (31.1)	327 (14.2)		
**Baseline FI, x̄ (SD)**	0.14 (0.10)	0.10 (0.05)	0.19 (0.07)	423.633†	<0.001
**Baseline FI level**				1819.019	<0.001
Robust	1939 (54.3)	1633 (45.7)	0 (0.0)		
Prefrail	2259 (55.1)	1057 (25.8)	782 (19.1)		
Frail	913 (64.2)	0 (0.0)	510 (35.8)		

FI – frailty index, SD – standard deviation, SVI – social vulnerability index, Q – quartile, x̄ – mean, χ^2^ – chi square

*All values are presented as numbers (percentages) unless specified otherwise.

†Values present F-statistic.

We analysed the impact of SVI on frailty transition status among the older population across different levels of frailty severity. We conducted a multivariate logistic regression analysis using frailty transition as the dependent variable and SVI as the independent variable, adjusting for age, gender, ethnicity, place of residence, and education level. There was no significant multicollinearity in SVI (VIF = 1.3), age (VIF = 2.1), gender (VIF = 1.2), ethnicity (VIF = 1.4), residence (VIF = 1.5), or education (VIF = 1.9), and the lowest tolerance value was 0.67. Additionally, no significant interaction effects were observed among the independent variables (*P* > 0.05). The logistic regression results indicated that an SVI increase was associated with an increased risk of worsening frailty conditions in robust (odds ratio (OR) = 1.017; *P* < 0.05) and prefrail (OR = 1.021; *P* < 0.05) older individuals (**Table 3**). However, the SVI increase was not associated with improvement in frailty status among prefrail and frail individuals. Further analysis by gender revealed that age was only associated with the risk of worsening frailty conditions in robust and prefrail older women and influenced frailty transition status in older men across different levels of frailty severity. Place of residence affected frailty transitions during follow-up only among frail older women, while it influenced worsening of frailty in robust older men and improvement in frailty in prefrail and frail older men. An SVI increase was associated with an elevated risk of worsening frailty conditions in robust (OR = 1.014; *P* < 0.05) and prefrail (OR = 1.025; *P* < 0.05) women and in robust (OR = 1.020; *P* < 0.05) and prefrail (OR = 1.017; *P* < 0.05) men, but had no impact on those who were already frail. This also suggests that social factors have a greater impact on robust and prefrail older individuals compared to those who are frail. The sensitivity analysis indicated that the association between SVI and frailty transition remained significant for robust (OR = 1.018; *P* < 0.05) and prefrail (OR = 1.023; *P* < 0.05) individuals (Table S3 in the **Online Supplementary Document**). Stratified analyses by gender revealed that women were more sensitive to the adjustments, while place of residence had no significant impact on frailty transition in older women, but did affect frailty transitions in older men.

**Table 3:** Logistic regression for SVI’s effect on frailty transition in the older adults

	Increasing age		Male gender		Ethnic minority		Rural residence		Higher education		Increasing SVI	
	**OR (95% CI)**	***P*-value**	**OR (95% CI)**	***P*-value**	**OR (95% CI)**	***P*-value**	**OR (95% CI)**	***P*-value**	**OR (95% CI)**	***P*-value**	**OR (95% CI)**	***P*-value**
**Total**												
Robust worsening	1.394 (1.249, 1.555)	<0.001	0.616 (0.536, 0.707)	<0.001	0.829 (0.568, 1.209)	0.330	1.195 (1.042, 1.371)	0.011	0.877 (0.736, 1.045)	0.142	1.017 (1.010, 1.024)	<0.001
Prefrail worsening	1.395 (1.255, 1.550)	<0.001	0.769 (0.660, 0.895)	0.001	0.906 (0.632, 1.298)	0.589	1.010 (0.869, 1.173)	0.900	0.814 (0.683, 1.970)	0.061	1.021 (1.013, 1.029)	<0.001
Prefrail improvement	0.842 (0.744, 0.952)	0.006	1.436 (1.216, 1.697)	<0.001	1.028 (0.698, 1.515)	0.888	0.760 (0.644, 0.899)	0.001	1.016 (0.811, 1.196)	0.878	0.995 (0.986, 1.003)	0.234
Frail improvement	0.801 (0.687, 0.934)	0.005	1.079 (0.857, 1.359)	0.517	2.080 (0.731, 3.515)	0.066	0.656 (0.524, 0.822)	<0.001	1.044 (0.801, 1.361)	0.748	0.996 (0.984, 1.008)	0.505
**Women**												
Robust worsening	1.198 (1.005, 1.428)	0.044			0.687 (0.365, 1.291)	0.243	1.182 (0.957, 1.460)	0.120	0.960 (0.736, 1.252)	0.763	1.014 (1.003, 1.025)	0.013
Prefrail worsening	1.273 (1.102, 1.471)	0.001			0.976 (0.606, 1.572)	0.921	1.036 (0.775, 1.152)	0.578	0.840 (0.657, 1.074)	0.165	1.025 (1.015, 1.036)	<0.001
Prefrail improvement	0.874 (0.723, 1.056)	0.162			1.265 (0.727, 2.202)	0.405	0.913 (0.714, 1.168)	0.472	1.011 (0.564, 1.062)	0.113	0.998 (0.990, 1.016)	0.694
Frail improvement	0.885 (0.722, 1.084)	0.237			1.504 (0.689, 3.281)	0.306	0.734 (0.550, 0.979)	0.035	1.105 (0.579, 1.172)	0.282	0.996 (0.988, 1.018)	0.674
**Men**												
Robust worsening	1.542 (1.340, 1.774)	0.001			0.934 (0.584, 1.491)	0.774	1.213 (1.012, 1.455)	0.037	0.836 (0.660, 1.058)	0.136	1.020 (1.010, 1.029)	<0.001
Prefrail worsening	1.556 (1.329, 1.822)	0.001			0.817 (0.469, 1.424)	0.477	1.112 (0.882, 1.403)	0.370	0.789 (0.609, 1.022)	0.072	1.017 (1.005, 1.029)	0.005
Prefrail improvement	0.846 (0.716, 0.999)	0.048			1.013 (0.496, 1.464)	0.562	0.654 (0.519, 0.822)	<0.001	1.122 (0.878, 1.433)	0.358	0.989 (0.978, 1.001)	0.064
Frail improvement	0.725 (0.569, 0.925)	0.010			2.675 (0.886, 5.563)	0.068	0.552 (0.383, 0.796)	0.001	1.367 (0.907, 2.061)	0.135	0.984 (0.966, 1.004)	0.111

CI – confidence interval, OR – odds ratio, SVI – social vulnerability index

## DISCUSSION

We provide a unique prospective cohort analysis of social frailty and physical frailty among older adults in Chinese communities. While the FI and SVI methods have been previously applied and validated in studies involving older adults in China [24,25], we further confirm their relevance and applicability. We explored the influence of social frailty on frailty transitions, highlighting the applicability and ongoing validation of these methods in the Chinese context. The FI model, widely used in routine care and community settings, is the most effective tool for assessing frailty in older adults, as it quantifies frailty through the accumulation of health deficits, providing a broader and more effective evaluation than other methods [26,27]. Our use of SVI to assess social frailty aligns with previous studies, confirming its reliability and robustness in understanding how social factors influence health outcomes in older adults [28–30]. Our findings indicate that both social and physical frailty are closely linked, with a moderate positive correlation. Previous research has similarly demonstrated that social frailty often contributes to the development of physical frailty, with the deterioration of social networks and support systems further accelerating the decline in physical health [31]. This indicates that social frailty is not only a key predictor of physical frailty but may also precede it, accelerating the transition between frailty stages. Concurrently, older adults with physical frailty often experience declines in daily living activities, sensory abilities (*e.g.* hearing and vision), and cognitive function. These deteriorations, in turn, affect their social interactions and participation, further contributing to social frailty. This underscores the interrelated nature of social and physical frailty, with each reinforcing the other in a feedback loop.

We found that frailty, as measured by the FI, increases exponentially with age, both at baseline in 2017 and at follow-up in 2019. The average annual growth rate of FI values in 2019 was higher than in 2017, suggesting that the accumulation of health deficits accelerates as individuals age. Additionally, older women consistently exhibited higher levels of frailty compared to men at all age groups, which is in line with our previous findings [24,32]. Women tend to accumulate health deficits at a faster rate than men, likely due to hormonal changes such as the decline in estrogen levels after menopause. This reduction in estrogen affects muscle function, increases fatigue, and accelerates overall frailty [33]. In contrast, frailty in older men is more closely associated with androgen levels, and higher levels of free testosterone and dihydrotestosterone have been shown to reduce the risk of frailty [34]. Thus, gender differences in frailty may be partly attributed to hormonal changes that influence both physical and psychological symptoms in ageing individuals.

Although some older individuals did not exhibit physical frailty (*i.e.* FI = 0), all individuals demonstrated some degree of social vulnerability. This finding is supported by Andrew et al.’s study [21], which suggests that, with advancing age, older adults experience a decline in social support, limited social participation, and increased social isolation. These challenges often arise from factors such as the loss of family members or friends, declining health (*e.g.* illness and cognitive-psychological disorders), or changes in living environments (*e.g.* moving into nursing homes or other long-term care facilities). Across all age groups, older women exhibited higher levels of social frailty than men, which is consistent with the existing literature [10,35–37]. In the Chinese context, women's greater vulnerability may be attributed to their primary roles in household management and caregiving, which often leave them with less time for leisure activities and social engagement [38,39]. Additionally, women’s greater vulnerability in living situations, particularly widowhood and living alone, appears to contribute to their higher levels of overall social vulnerability [10]. Consequently, older women are more susceptible to social frailty than men. To mitigate this, older women should be encouraged to maintain a positive outlook and prioritise leisure and social activities. Moreover, family members and community health care workers should remain vigilant in supporting their physical, psychological, and social well-being, intervening promptly when needed to prevent further social isolation and frailty.

Frailty is a dynamic condition, and we found that over a two-year follow-up, most older adults (56.2%) maintained stable frailty status, while 29.6% worsened and 14.2% improved. The progression to a more severe frailty state was more common than improvement, and transitions between adjacent frailty stages were more frequent than those across multiple stages. Only 4.4% of frail individuals returned to a robust state, which is consistent with other studies. For example, the Survey of Health, Ageing and Retirement in Europe study found that, after two years, 61.8% of participants maintained their frailty status, 22.1% worsened, and 16.1% improved [40]. A similar study in Germany found that frailty deterioration was more common than improvement, with only 0–0.3% of individuals recovering to a robust state after an average follow-up of 2.1 years [41]. We found that men, particularly in the robust and prefrail groups, were more likely to maintain or improve their frailty status compared to women, who were more likely to experience worsening frailty. Among frail individuals, however, there were no significant gender differences, which aligns with the findings of Lorenzo-López et al. [42] and Ye et al. [43]. These results suggest that men have a greater potential for improvement in the earlier stages of frailty, while women may face a greater burden due to gender-related physical and mental health disadvantages. We found that the risk of worsening frailty increases with age. As individuals age, the accumulation of health deficits and the decline of organ function make them more susceptible to frailty. Additionally, older adults are more likely to experience psychological issues, reduced social participation, and changes in their living environment, all of which accelerate frailty progression. These findings highlight the importance of early interventions, especially in older adults who are robust or prefrail, to prevent further deterioration.

We also found that an increase in social frailty is a significant risk factor for the worsening of frailty conditions, particularly among robust and prefrail older adults, with no correlation to improvements in frailty status. While the OR for the association between SVI and frailty transition was modest, it is important to consider the significance of even small changes in SVI. In the context of ageing populations, small yet consistent changes in social vulnerability can cumulatively affect frailty transitions over time. This highlights the potential for even modest shifts in SVI to significantly affect frailty outcomes, especially when compounded across large populations or over extended periods. Furthemore, we found that age is linked to an increased risk of frailty deterioration only among robust and prefrail older women, while frailty transitions in men occur across all levels of frailty. This highlights that both robust and prefrail men and women are at higher risk of worsening frailty as they age, but younger older men are more likely to show improvement. Therefore, early frailty assessments and personalised intervention plans, particularly for younger older individuals – especially men – could help reverse or delay frailty progression more effectively. Additionally, older rural adults face heightened risks of frailty deterioration. As in China's social context, older adults in rural areas often face greater challenges, including limited access to health care, social isolation, restricted economic support, and fewer social welfare services. These factors exacerbate frailty, especially in older men. As such, rural populations require targeted interventions to improve social support, increase participation in social activities, and enhance health care access. By focusing on these factors, interventions can be more effective in preventing frailty deterioration, particularly in the early stages, and contribute to healthier ageing in both rural and urban settings.

This study has several limitations. First, the variables we used to assess physical frailty and social frailty are based on self-reported data from older individuals, which could introduce recall and social desirability bias. However, self-perceived frailty may be more closely related to their health conditions than objective measures [43]. Second, due to the feasibility of the SSAPUR survey, specific objective indicators that may affect frailty, such as chronic inflammation (*e.g.* C-reactive protein and interleukin-6) or genetic predisposition, as well as some qualitative data to provide a deeper understanding of the social frailty drivers (*e.g.* loneliness, poverty, and other socio-emotional factors), were not included in the questionnaire. Moreover, while we adjusted for key confounders, residual confounding (*e.g.* genetic predisposition and early-life adversity) and potential reverse causality still limit the causal interpretation of our findings. Future research should broaden data collection to include biomarkers, genetic information, and detailed psychosocial metrics to better address residual confounding. Additionally, employing intervention designs and longitudinal assessments will be essential to interpret the causal pathways between social and physical frailty.

## CONCLUSIONS

We found that, as age increased, frailty deterioration among older adults was more common than improvement, and higher social frailty was associated with an increased risk of frailty deterioration. This impact was more pronounced for robust and prefrail older adults compared to those who were already in a frail state. Given the unique socioeconomic and health care challenges in China, interventions aimed at addressing social frailty should prioritise robust and prefrail individuals.

To effectively mitigate frailty progression, policies and medical interventions need to take a comprehensive approach, emphasising social engagement and encouraging widespread participation in community activities. Early identification of social frailty and targeted interventions can delay its onset and reduce its impact on the older population. These efforts are particularly vital in the Chinese context, where demographic shifts and regional disparities present additional challenges in maintaining the health and well-being of older adults.

## Additional material


Online Supplementary Document
