# Supplementary material for: Impact of social vulnerability on frailty transition among older adults in China: a national two-year cohort study
Source: J Glob Health. 2025 Nov 7;15:04284. doi: 10.7189/jogh.15.04284 (PMC12591508; doi:10.7189/jogh.15.04284)

Supplement to: Shi J, Tao Y, Cen Y, Gao C, Zhang L, Li S, Li Y, Sang B, Liu X, Ma Q, Zeng X, Li J, Liu D. Impact of social vulnerability on frailty transition among older adults in China: a national two-year cohort study. J Glob Health. 2025;15:04284.

Table S1. Variables and calculation of frailty index.

Frailty index	Cut-off
**ADL**	
1. Feeding	Yes =0, with difficulty =0.5, need help =1
2. Getting dressed	Yes =0, with difficulty =0.5, need help =1
3. Using the toilet	Yes =0, with difficulty =0.5, need help =1
4. Getting in and out of bed	Yes =0, with difficulty =0.5, need help =1
5. Walking indoors	Yes =0, with difficulty =0.5, need help =1
6. Bathing	Yes =0, with difficulty =0.5, need help =1
**Chronic diseases**	
7. Glaucoma/cataracts	Yes = 1, no = 0
8. Hypertension	Yes = 1, no = 0
9. Diabetes	Yes = 1, no = 0
10.Cardiovascular and cerebrovascular diseases	Yes = 1, no = 0
11.Gastric diseases	Yes = 1, no = 0
12. Osteoarthritis	Yes = 1, no = 0
13. Chronic lung disease	Yes = 1, no = 0
14.Asthma	Yes = 1, no = 0
15. Cancer	Yes = 1, no = 0
16.Reproductive system disorders	Yes = 1, no = 0
17.Other diseases	Yes = 1, no = 0
**Geriatric symptoms**	

18.Urinary incontinence	Yes = 1, no = 0
19. Faecal incontinence	Yes = 1, no = 0
20.Fall history	Yes = 1, no = 0
21.Visual impairment	Normal = 0, mildly impaired = 0.25, moderately impaired = 0.5, severely impaired = 0.75, almost blind = 1
22.Hearing impairment	Normal =0, mildly impaired =0.5, severely impaired =1
**Health status and emotion**	
23. How do you feel about your health status?	Excellent =0, good =0.25, fair =0.5, not good =0.75, poor =1
24. Do you currently need someone to take care of you in your daily life?	Yes = 1,no = 0
25.Do you feel lonely?	Often = 1, sometimes = 0.5, never = 0
26. Howhappy do you feel?	Very happy = 0, happy = 0.25, moderately happy = 0.5, unhappy = 0.75, very unhappy = 1
**Use of assistive devices**	
27.Hearing aids	Yes = 1, no = 0
28.Dentures	Yes = 1, no = 0
29.Crutches	Yes = 1, no = 0
30.Wheelchair	Yes = 1, no = 0
31.Adult diapers/pads	Yes = 1, no = 0

Note: ADL: Activities of daily living

Table S2. Demographics and social vulnerability-related factors among older adults with different levels of frailty

Variables	All					Women					Men				
	Total	Robust	Prefrail	Frail	*P*-value	Total	Robust	Prefrail	Frail	*P*-value	Total	Robust	Prefrail	Frail	*P*-value
N	9093	3572	4098	1423		4495	1481	2157	857		4598	2091	1941	566	
**Age** (x±s)	71.2 ±7.0	69.3±6.1	71.7±6.9	74.2±7.8	<0.001	71.5± 7.2	69.2±6.2	71.9±7.1	74.4±7.9	<0.001	70.9 ±6.8	69.4±6 .1	71.6±6. 8	74.0± 7.7	<0.001
**Agegroup**					<0.001					<0.001					<0.001
60-69	4499	48.5%	41.2%	10.3%		2169	42.7%	44.5%	12.7%		2330	53.8%	38.1%	8.1%	
70-79	3247	33.4%	49.0%	17.6%		1597	26.9%	52.2%	20.9%		1650	39.7%	45.8%	14.5%	
80+	1347	22.8%	48.6%	28.7%		729	17.1%	49.0%	33.9%		618	29.4%	48.1%	22.5%	
**Gender**					<0.001										
Women	4495	32.9%	48.0%	19.1%											
Men	4598	45.5%	42.2%	12.3%											
**Ethnicity**					0.034					0.017					0.114
Han	8720	39.6%	44.8%	15.6%		4326	33.3%	47.6%	19.2%		4394	45.7%	42.1%	12.2%	
Minority	373	33.0%	50.7%	16.4%		169	24.9%	58.6%	16.6%		204	39.7%	44.1%	16.2%	
**Residence**					<0.001					<0.001					0.071
Urban	4656	41.2%	44.1%	14.7%		2377	35.5%	46.9%	17.7%		2279	47.1%	41.2%	11.7%	
Rural	4437	37.3%	46.1%	16.6%		2118	30.1%	49.2%	20.6%		2319	43.9%	43.2%	12.9%	
**Education**					<0.001					<0.001					<0.001
No formal education	6633	43.3%	43.3%	13.4%		2851	37.7%	46.5%	15.7%		3782	47.6%	40.9%	11.6%	
≤Junior high school	2273	27.8%	49.6%	22.6%		1604	24.3%	50.6%	25.1%		669	36.3%	47.1%	16.6%	
≥Senior high school	187	34.2%	53.5%	12.3%		40	37.5%	47.5%	15.0%		123	33.3%	55.1%	11.6%	
**Social**															

**frailty-related factors**															
**Marital status**					<0.001					<0.001					<0.001
Married	6633	43.3%	43.3%	13.4%		2851	37.7%	46.5%	15.7%		3782	47.6%	40.9%	11.6%	
Others[a]	2399	28.2%	49.9%	22.0%		1607	24.4%	50.6%	25.0%		792	35.9%	48.4%	15.8%	
**Living alone**	1480	28.9%	50.8%	20.3%	<0.001	854	23.7%	52.6%	23.8%	<0.001	626	36.1%	48.4%	15.5%	<0.001
**Medicare coverage**	9021	39.3%	45.1%	15.6%	0.667	4456	33.0%	48.0%	19.0%	0.499	4565	45.5%	42.2%	12.3%	0.924
**Medical reimbursement**					<0.001					<0.001					<0.001
Very convenient	4088	41.1%	44.7%	14.2%		2009	35.0%	48.1%	16.9%		2079	47.0%	41.4%	11.5%	
Relatively convenient	2539	37.6%	45.6%	16.8%		1266	30.3%	48.3%	21.5%		1273	44.9%	43.0%	12.2%	
Fair	786	31.9%	49.5%	18.6%		397	27.2%	52.4%	20.4%		389	36.8%	46.5%	16.7%	
Relatively inconvenient	222	24.3%	48.6%	27.0%		109	18.3%	52.3%	29.4%		113	30.1%	45.1%	24.8%	
Very inconvenient	94	23.4%	51.1%	25.5%		45	17.8%	48.9%	33.3%		49	28.6%	53.1%	18.4%	
**Support during illness**	898	3.2%	31.8%	64.9%	<0.001	516	2.9%	30.6%	66.5%	<0.001	382	3.7%	33.5%	62.8%	<0.001
**Still in paid employment**	917	64.2%	32.2%	3.6%	<0.001	263	55.1%	38.8%	6.1%	<0.001	654	67.9%	29.5%	2.6%	<0.001
**Receiving pension**	3092	49.9%	40.8%	9.2%	<0.001	1428	43.3%	44.7%	12.0%	<0.001	1664	55.6%	37.5%	6.9%	<0.001
**Home**	6572	42.5%	44.2%	13.3%	<0.001	3033	36.7%	46.9%	16.4%	<0.001	3539	47.4%	41.9%	10.7%	<0.001

**ownership**															
**Economic status**					<0.001					<0.001					<0.001
Very wealthy	189	75.7%	20.1%	4.2%		80	73.8%	21.3%	5.0%		109	77.1%	19.3%	3.7%	
Fairly wealthy	1543	56.3%	37.1%	6.5%		712	49.2%	43.0%	7.9%		831	62.5%	32.1%	5.4%	
Just enough	5389	41.1%	45.9%	13.0%		2709	34.3%	49.3%	16.4%		2680	48.0%	42.4%	9.6%	
Somewhat difficult	1646	18.0%	52.7%	29.3%		832	14.3%	52.0%	33.7%		814	21.7%	53.4%	24.8%	
Very difficult	302	12.6%	44.7%	42.7%		152	11.8%	40.8%	47.4%		150	13.3%	48.7%	38.0%	
**Time of the house built**					<0.001					0.018					<0.001
After 2000s	3566	40.6%	44.6%	14.8%		1768	33.3%	48.5%	18.3%		1798	47.8%	40.7%	11.5%	
In the 1990’ s	2594	41.6%	44.3%	14.0%		1286	35.5%	47.0%	17.5%		1308	47.7%	41.7%	10.6%	
1970s-1980s	2396	36.6%	45.7%	17.6%		1166	31.0%	48.3%	20.7%		1230	42.0%	43.3%	14.7%	
Prior to 1950s-1960s	526	30.2%	49.8%	20.0%		270	26.7%	48.9%	24.4%		256	34.0%	50.8%	15.2%	
**Housing area（m^2）**					<0.001					<0.001					<0.001
≥145	1990	44.8%	42.0%	13.2%		996	37.8%	45.5%	16.8%		994	51.9%	38.4%	9.7%	
91-144	2595	40.9%	45.4%	13.7%		1313	35.5%	47.9%	16.6%		1282	46.4%	42.9%	10.7%	
61-90	2294	38.1%	45.0%	16.9%		1073	31.4%	48.1%	20.5%		1221	44.0%	42.3%	13.8%	
<60	2111	32.8%	48.1%	19.1%		1068	26.5%	50.9%	22.6%		1043	39.3%	45.2%	15.5%	
**Having private living rooms**	8663	39.6%	44.9%	15.5%	0.023	4278	33.2%	47.8%	19.0%	0.250	4385	45.8%	42.0%	12.2%	0.088

**No issues with living conditions**	4846	47.7%	42.1%	10.2%	<0.001	2325	41.2%	45.9%	12.8%	<0.001	2521	53.7%	38.5%	7.8%	<0.001
**Satisfaction with living conditions**					<0.001					<0.001					<0.001
Satisfied	5341	44.5%	42.6%	12.8%		2597	38.0%	46.4%	15.6%		2744	50.7%	39.1%	10.2%	
Average	2899	34.8%	47.9%	17.3%		1475	28.5%	50.2%	21.3%		1424	41.3%	45.6%	13.1%	
Not satisfied	799	21.2%	50.1%	28.8%		401	16.7%	49.9%	33.4%		398	25.6%	50.3%	24.1%	
**Public welfare participation**	4874	43.9%	44.3%	11.9%	<0.001	2258	37.2%	47.6%	15.1%	<0.001	2616	49.6%	41.4%	9.0%	<0.001
**Senior associations participation**	1595	47.1%	41.9%	11.0%	<0.001	762	38.8%	46.6%	14.6%	<0.001	833	54.6%	37.6%	7.8%	<0.001
**Helping seniors in need**	7268	41.5%	44.5%	14.0%	<0.001	3534	34.8%	47.8%	17.3%	<0.001	3734	47.8%	41.3%	10.8%	<0.001
**Legal rights protected**	8539	39.9%	45.0%	15.1%	<0.001	4224	33.3%	48.3%	18.3%	<0.001	4315	46.3%	41.7%	12.0%	<0.001
**Recreational participation**	8543	40.4%	45.1%	14.5%	<0.001	4122	34.4%	48.2%	17.4%	<0.001	4421	46.0%	42.3%	11.8%	<0.001
**Regular internet access**	682	53.8%	40.0%	6.2%	<0.001	255	45.1%	48.6%	6.3%	<0.001	427	59.0%	34.9%	6.1%	<0.001
**Online education participation**	246	47.6%	43.5%	8.9%	0.003	118	44.1%	47.5%	8.5%	0.003	128	50.8%	39.8%	9.4%	0.391
**SVI**	0.40±0.12	0.36±0.11	0.42±0.11	0.47±0.11	<0.001	0.42±0.12	0.37±0.11	0.43±0.11	0.48±0.11	<0.001	0.38±0.11	0.35±0.11	0.40±0.11	0.45±0.10	<0.001

**SVI level**					<0.001					<0.001					<0.001
Q1	2314	58.7%	35.8%	5.4%		943	51.2%	40.5%	8.3%		1371	63.9%	32.6%	3.5%	
Q2	2280	43.8%	44.3%	11.9%		1023	39.7%	47.4%	12.9%		1257	47.2%	41.8%	11.1%	
Q3	2200	32.2%	49.8%	18.0%		1168	29.6%	50.3%	20.0%		1032	35.2%	49.2%	15.6%	
Q4	2299	22.0%	50.6%	27.4%		1361	18.1%	51.6%	30.3%		938	27.6%	49.1%	23.2%	

Note: [a]Other marital status includes widowed, divorced or never married; SVI: social vulnerability index

Table S3. Sensitivity analysis of SVI's effect on frailty transition in the older adults (*OR*(95%*CI*))

Variables	Total				Women				Men			
	Robust worsening	Prefrail worsening	Prefrail improvement	Frail improvement	Robust worsening	Prefrail worsening	Prefrail improvement	Frail improvement	Robust worsening	Prefrail worsening	Prefrail improvement	Frail improvement
Agegroup (Increasing)	1.268* (1.134, 1.497)	1.273* (1.152, 1.465)	0.896* (0.712,0.971)	0.792* (0.667,0.925)	1.164* (1.009,1.502)	1.295* (1.136,1.653)	0.779(0.534,1.089)	0.663 (0.518,1.006)	1.439* (1.261,1.705)	1.529* (1.269, 1.895)	0.751* (0.624, 0.987)	0.634* (0.491,0.936)
Gender (Male)	0.566* (0.437,0.729)	0.697* (0.591,0.866)	1.451* (1.267,1.776)	1.110 (0.912,1.396)								
Ethnicity (Minority)	0.779 (0.496,1.312)	0.885 (0.601,1.339)	1.104(0.778,1.596)	1.986 (0.656,2.910)	0.666 (0.312,1.334)	0.899 (0.674.1.669)	1.311(0.810,2.354)	1.603 (0.691,3.332)	0.814 (0.433,1.327)	0.808 (0.451, 1.403)	1.022 (0.501, 1.527)	3.001 (0.895,5.891)
Residence place(Rural)	1.187* (1.021,1.296)	1.103 (0.875,1.232)	0.691* (0.549,0.775)	0.679* (0.531,0.901)	1.177 (0.815,1.339)	1.051 (0.803,1.166)	0.899 (0.682,1.475)	0.742 (0.556,1.214)	1.229* (1.056,1.553)	1.239 (0.896, 1.491)	0.737* (0.526, 0.887)	0.493* (0.325,0.717)
Education (Higher)	0.864 (0.754,1.067)	0.779 (0.621,1.856)	1.028 (0.892,2.025)	1.043 (0.793,1.352)	0.897 (0.712,1.249)	0.842 (0.671,1.142)	1.034 (0.692,1.153)	1.111 (0.625,1.213)	0.991 (0.724,1.132)	0.781(0.623,1.119)	1.130 (0.902, 1.456)	1.326 (0.897,1.928)

SVI(Increasing)	1.018* (1.012,1.031)	1.023*(1.014,1.323)	0.996 (0.983,1.004)	0.995 (0.982,1.008)	1.016* (1.005,1.023)	1.029*(1.014,1.052)	0.997 (0.991,1.018)	0.989 (0.963,1.020)	1.023* (1.001,1.036)	1.018* (1.004, 1.026)	0.986 (0.954, 1.003)	0.979 (0.954.1.008)

Note: SVI: Social Vulnerability Index; CI: Confidence interval; *indicate statistical significance

Figure S1. SVI values among different age groups.

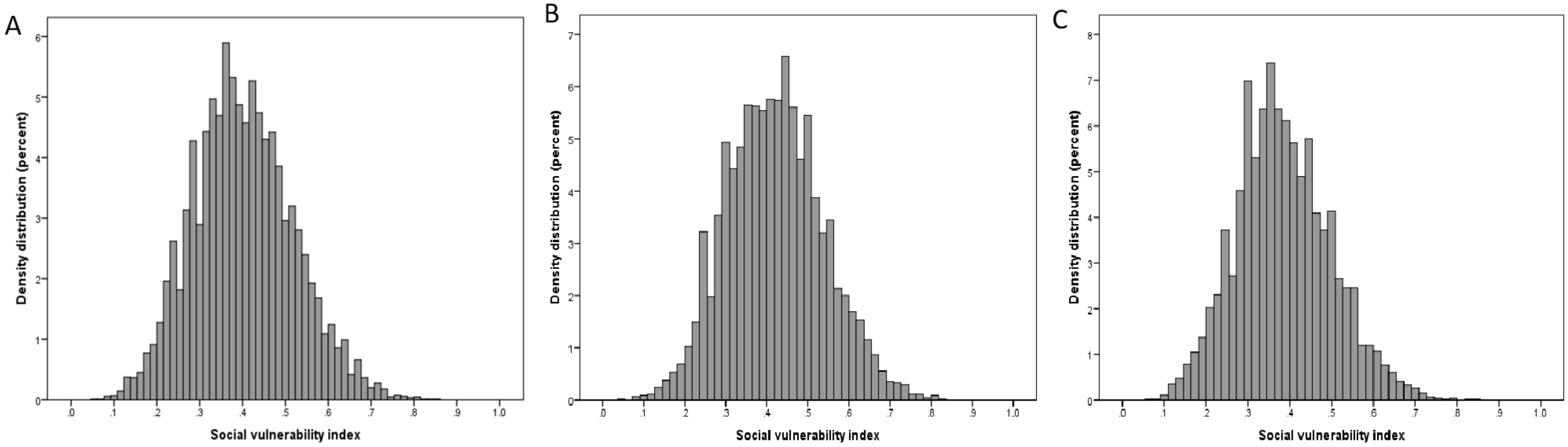

Figure S2. Frailty progression.

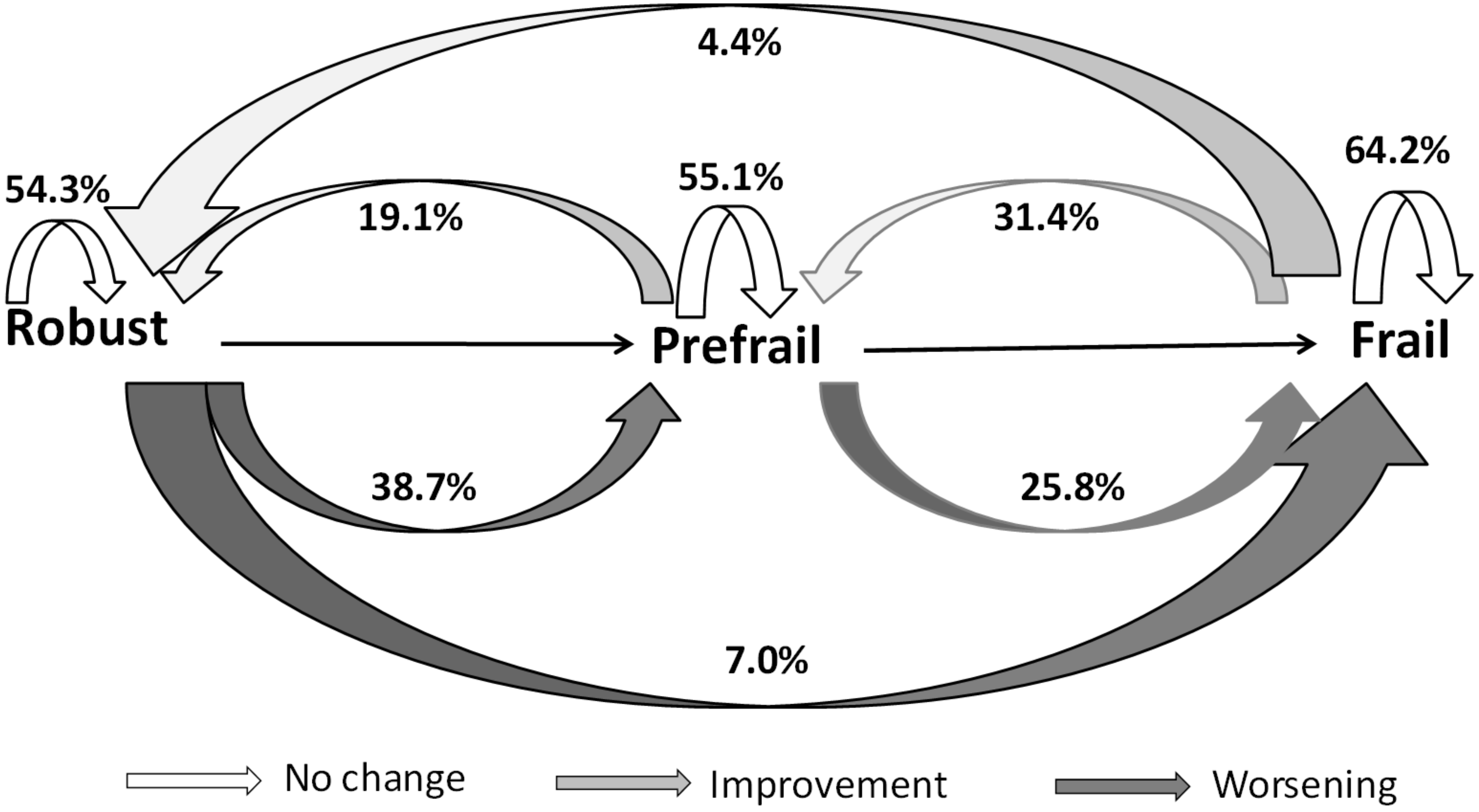

Supplement: Online Supplementary Document [file jogh-15-04284-s001.pdf]
